# A novel *WFS1 *mutation in a family with dominant low frequency sensorineural hearing loss with normal VEMP and EcochG findings

**DOI:** 10.1186/1471-2350-9-48

**Published:** 2008-06-02

**Authors:** Naomi F Bramhall, Jeremy C Kallman, Aimee M Verrall, Valerie A Street

**Affiliations:** 1 Department of Speech and Hearing Sciences, University of Washington, Seattle, USA; 2 V.M. Bloedel Hearing Research Center, Otolaryngology – HNS Department, University of Washington, Seattle, USA

## Abstract

**Background:**

Low frequency sensorineural hearing loss (LFSNHL) is an uncommon clinical finding. Mutations within three different identified genes (*DIAPH1, MYO7A*, and *WFS1*) are known to cause LFSNHL. The majority of hereditary LFSNHL is associated with heterozygous mutations in the *WFS1 *gene (wolframin protein). The goal of this study was to use genetic analysis to determine if a small American family's hereditary LFSNHL is linked to a mutation in the *WFS1 *gene and to use VEMP and EcochG testing to further characterize the family's audiovestibular phenotype.

**Methods:**

The clinical phenotype of the American family was characterized by audiologic testing, vestibular evoked myogenic potentials (VEMP), and electrocochleography (EcochG) evaluation. Genetic characterization was performed by microsatellite analysis and direct sequencing of *WFS1 *for mutation detection.

**Results:**

Sequence analysis of the *WFS1 *gene revealed a novel heterozygous mutation at c.2054G>C predicting a p.R685P amino acid substitution in wolframin. The c.2054G>C mutation segregates faithfully with hearing loss in the family and is absent in 230 control chromosomes. The p.R685 residue is located within the hydrophilic C-terminus of wolframin and is conserved across species. The VEMP and EcochG findings were normal in individuals segregating the *WFS1 *c.2054G>C mutation.

**Conclusion:**

We discovered a novel heterozygous missense mutation in exon 8 of *WFS1 *predicting a p.R685P amino acid substitution that is likely to underlie the LFSNHL phenotype in the American family. For the first time, we describe VEMP and EcochG findings for individuals segregating a heterozygous *WFS1 *mutation.

## Background

Hearing loss is a common disorder affecting the communication abilities of more than 28 million Americans. A variety of etiologies for hearing loss have been identified including aging, noise exposure, ototoxic medications, and genetics. Over 90 different genetic loci have been linked to hereditary auditory impairment [1]. The majority of these loci are associated with high frequency hearing loss or a deficit affecting all frequencies, with only four loci linked to low frequency hearing loss. It is not well understood why autosomal dominant mutations at these four loci cause low frequency hearing loss as opposed to reduced sensitivity in the higher frequencies.

Non-syndromic autosomal dominant low frequency sensorineural hearing loss (LFSNHL) has been mapped to the *DFNA1, DFNA11, DFNA54*, and *DFNA6/14/38 *loci. These four loci are described by the letters *DFN*, which stand for deafness, and the letter *A *to indicate autosomal dominant inheritance. The loci are labeled with a number indicating the order in which they were discovered. *DFNA1*, the first identified locus for LFSNHL, is associated with a mutation in the *DIAPH1 *gene (homologue of *Drosophila diaphanous*) and has been reported in only a single family [2]. *DFNA11 *represents a second LFSNHL locus, where a heterozygous mutation in myosin VIIA (*MYO7A*) found in only a single family leads to non-syndromic LFSNHL [3]. *DFNA54 *was mapped to LFSNHL in one family, but the gene has not yet been identified [4]. The fourth and most common locus for LFSNHL is *DFNA6/14/38*, which results from heterozygous mutations in the Wolfram syndrome type 1 gene (*WFS1*) [5-7].

The hearing loss associated with autosomal dominant mutations in *WFS1 *is primarily bilateral and symmetric. The age of onset of hearing loss appears to occur before age 10 and initially affects 250, 500 and 1000 Hz [8]. The mild hearing loss in the low frequencies seen in young children generally progresses to a moderate hearing loss in the low and mid frequencies by the second decade of life and then to a moderate to severe loss across the frequency range after age 40 [7]. Homozygous mutations in *WFS1 *have been linked to the recessively inherited Wolfram syndrome, a syndrome whose features include diabetes insipidus, diabetes mellitus, optic atrophy and sensorineural hearing loss in the high frequencies [9].

*WFS1 *encodes for wolframin, an 890 amino acid protein of unknown function, which has been localized to the endoplasmic reticulum [10]. Wolframin is predicted to have nine transmembrane spanning domains with a hydrophilic N- and C-terminus [11]. Wolframin is expressed in the mouse inner ear throughout postnatal development (P1, 7, 14, 35) in a variety of cell types including outer and inner hair cells, support cells, spiral ganglion neurons and vestibular hair cells [12]. No wolframin expression gradient is observed between the apical and basal ends of the cochlea.

Despite high levels of wolframin expression in the vestibular hair cells [12], individuals with heterozygous *WFS1 *mutations and LFSNHL generally do not complain of vestibular difficulty [7,8,13-17]. Ocular motor, caloric and vestibular ocular reflex testing have not revealed any consistent vestibular dysfunction in patients with Wolfram's syndrome or individuals with heterozygous *WFS1 *mutations and LFSNHL [15,17-21]. Previous vestibular testing for individuals with heterozygous and homozygous *WFS1 *mutations has targeted the semicircular canals. To our knowledge, no clinical testing of otolith function such as the vestibular evoked myogenic potential (VEMP) has been completed in individuals with *WFS1 *mutations. In addition, the literature does not report electrocochleography (EcochG) findings for individuals with *WFS1 *mutations even though LFSNHL has also been associated with endolymphatic hydrops [22].

The goal of this study was to use genetic analysis to determine if a small American family's hereditary LFSNHL is linked to a mutation in the *WFS1 *gene and to use VEMP and EcochG testing to further characterize the family's audiovestibular phenotype.

## Methods

### Research subjects and controls

To facilitate microsatellite and sequence analysis, blood samples were collected by venipuncture from four unaffected and three affected family members and 115 predominantly Caucasian control subjects for high molecular weight DNA isolation using standard techniques. Control subjects were screened to ensure that they had normal hearing for their age as previously described [3]. Individuals gave written informed consent according to a protocol approved by the Institutional Review Board (IRB) of the University of Washington.

### Auditory assessment

Audiologic evaluations were either conducted as part of this study or obtained from previous test results that were released to the study. Audiograms were obtained by a certified audiologist for seven affected members of the family in either a sound treated booth or a quiet room during a family reunion. Immittance testing was used to evaluate middle-ear pressure, ear canal volume, and tympanic membrane mobility. Each study participant completed a hearing and balance questionnaire to assess his or her medical and noise-exposure history.

### VEMP and EcochG assessment

The VEMP and EcochG evaluations were conducted in the Otolaryngology – HNS Clinic testing suites at the University of Washington. Vestibular Evoked Myogenic Potential (VEMP) uses a high intensity auditory stimulus to elicit a neck muscle response for assessment of saccular function. Abnormal saccular function is indicated by the absence of the VEMP response. Person 20 was presented monaurally with 250 clicks at 5 Hz and 100 dB nHL. Potentials across the ventral neck muscles were recorded bilaterally using electromyography with surface electrodes. Electrocochleography (EcochG) measures an auditory evoked response generated by the cochlea and auditory nerve. The EcochG response consists of the cochlear microphonic, the summating potential (SP), and the action potential (AP). An increased SP/AP amplitude ratio is often used as a diagnostic indicator of endolymphatic hydrops and Meniere's disease. Persons 20 and 26 were tested using the Nicolet Spirit evoked potential system. Electrodes were placed on the forehead and tiptrodes inserted into the ear canals. Click stimuli were presented at a rate of 11.4 per second. Response waveforms were obtained for an alternating polarity stimulus over a 10 ms time window and for condensation and rarefaction stimuli over 5 ms. Responses were recorded with filter settings of 20–3000 Hz and averaged over 800–1000 sweeps.

### Microsatellite marker, DNA sequence, and protein bioinformatics analysis

Microsatellite markers near the *DFNA1*, *DFNA11*, *DFNA4/16/38 *and *DFNA54 *loci were analyzed in seven family members. PCR products were multiplexed and separated by capillary electrophoresis on an ABI PRISM 310 Genetic Analyzer (PE Biosystems, Foster City, CA). Amplification products were sized according to CEPH (Centre d'Etude du Polymorphisme Humain) control DNA (1347-02) and assigned allele numbers consistent with the CEPH designations [23]. The inheritance pattern of the hearing loss was assumed to be autosomal dominant and fully penetrant.

PCR primers for exons 1–8 and the immediate intron flanking regions were designed with the Primer 3 web-based program [24] and used to analyze the *WFS1 *gene in one unaffected and two affected family members (primer sequences available upon request). PCR incubation mixture, thermocycling conditions, and purification procedures for genomic amplification and sequencing were performed as described previously [25,26]. Electropherograms were analyzed using the CodonCode Aligner software package (CodonCode Corporation, Dedham, MA).

The C-terminus region of wolframin (amino acids 657–890) was analyzed using The PSIPRED Structure Prediction Server [27-32] and FoldIndex [33].

## Results

### Low-frequency hearing loss

**Figure 1:**
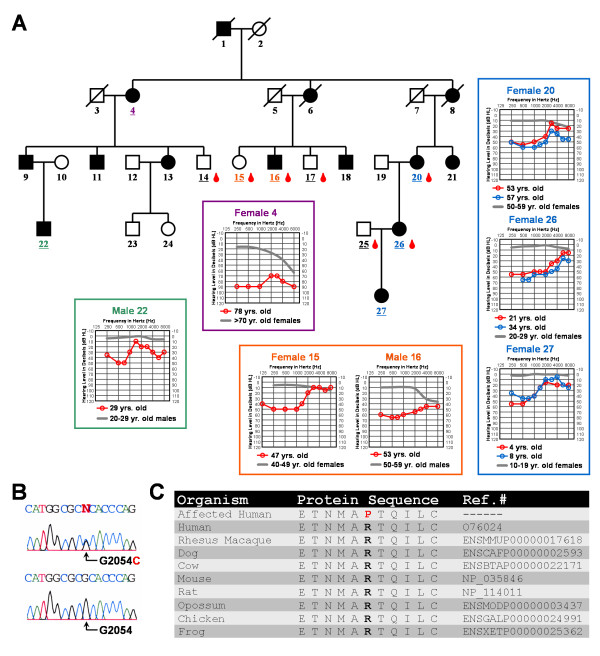
**Audiologic and genetic characterization of the low frequency hearing loss pedigree. (A) **Each individual in the pedigree is assigned a number. Underlined numbers indicate that auditory evaluations were performed for that person. Affected individuals are denoted by blackened symbols, males are denoted by squares, females are denoted by circles, and deceased persons are indicated by a diagonal line through the symbol. Blood drop symbols indicate individuals who donated a blood sample to the study. Symmetrical hearing loss was detected in all affected family members; therefore, only the right ear pure-tone air conduction thresholds are plotted on the audiograms. Frequency in hertz (Hz) is plotted on the x-axis and the hearing level in decibels (dB HL) on the y-axis. Plotted on each audiogram (gray line) are the average pure-tone air conduction thresholds for a person with normal hearing matched in age [39] to the family member. **(B) **Electropherograms showing a heterozygous c.2054G>C mutated genomic nucleotide sequence from an affected individual compared to a homozygous c.2054G unaffected family member. Nucleotide numbering starts with the ORF. **(C) **Protein alignment shows conservation of the p.R685 residue during evolution. The p.685P substitution in the pedigree is shown in red.

Affected family members demonstrated bilateral symmetric moderate or moderately-severe sensorineural hearing loss in the low frequencies, rising to normal or borderline normal at 4000 Hz and above (Fig. 1A). A moderate low frequency hearing loss found in a 4-year-old child suggests childhood onset of hearing loss (Fig. 1A). Longitudinal audiometric time points were available for females 20, 26, and 27 suggesting a slow rate of hearing loss progression in these three individuals (Fig. 1A). Speech audiometry results for two family members indicated speech discrimination ability within normal limits.

### Genetic analysis supports novel mutation in WFS1 exon 8

Microsatellite markers near the *DFNA1*, *DFNA11*, and *DFNA54 *loci did not cosegregate with hearing loss in the American pedigree. However, allele 6 (234 bp product) for marker *D4S432 *near the *DFNA4/16/38 *locus did cosegregate with hearing loss in the family, leading us to consider further the candidacy of the *WFS1 *gene by direct sequence analysis. Sequencing of *WFS1 *exon 8 revealed a c.2054G>C transversion (Fig. 1B) predicting a p.R685P amino acid substitution (Fig. 1C). Sequence analysis indicated the absence of this transversion in 230 control chromosomes. This heterozygous c.2054G>C change is consistent with the autosomal dominant inheritance observed for hearing loss in the family (Fig. 1A). The p.R685 residue is located within the hydrophilic C-terminus of wolframin and conserved across human, rhesus monkey, dog, cow, mouse, rat, opossum, chicken and frog (Fig. 1C).

### Protein bioinformatics predicts a folded Wolframin C-terminus

Conserved structural protein motifs have not been identified in the C-terminus region of wolframin nor has a solved protein structure for this region been published. In this report, the C-terminus region of wolframin was analyzed using The PSIPRED Structure Prediction Server and FoldIndex. These protein bioinformatics programs suggest that the C-terminal region of wolframin adopts a folded confirmation. It is likely that the p.R685 residue is exposed on the surface of this folded domain where it could facilitate protein-to-protein interactions.

### Normal VEMP, and EcochG findings

No balance problems were self-reported by the family members on the intake questionnaire. The VEMP was present bilaterally in person 20. The EcochG test generated an SP/AP ratio within the normal range for both persons 20 and 26.

## Discussion

We identified a small Caucasian American family with hereditary autosomal dominant LFSNHL likely caused by a novel heterozygous c.2054G>C *WFS1 *mutation. The absence of this heterozygous c.2054G>C DNA alteration in 230 control chromosomes supports the hypothesis that it represents a causative mutation, not a rare polymorphism. The c.2054G>C DNA alteration cosegregates faithfully with hearing loss in the family and predicts a non-conservative p.R685P amino acid substitution in the wolframin protein. The arginine amino acid residue at position 685 is found not only in humans, but also in the rhesus monkey, mouse, rat, dog, cow, opossum, chicken and frog. Conservation of p.R685 over evolutionary time suggests that altering this residue could have deleterious consequences for the wolframin protein. The p.R685P amino acid substitution may alter the ability of the C-terminus domain to adopt a folded conformation or may impede interaction with a protein binding partner. A recent report using a yeast two-hybrid system with a human brain cDNA library suggests that the C-terminal domain (amino acids 652–890) of the human *WFS1 *gene interacts with the Na^+^/K^+ ^ATPase β1 subunit [34]. If a similar association exists within the inner ear, perhaps the p.R685P substitution alters this protein-to-protein interaction.

Twenty-six other heterozygous *WFS1 *mutations linked to dominant low frequency sensorineural hearing loss have been described previously [35]. These known mutations include 25 point mutations and one small deletion, all found in the large exon 8 with the exception of two point mutations in exon 5. This wealth of mutational genetic heterogeneity within *WFS1 *will contribute useful information for uncovering the structure/function relationship of the wolframin protein in the auditory and vestibular system.

Previous vestibular testing focusing on semicircular canal function in individuals with *WFS1 *mutations yielded normal findings [15,17-21]. However, saccular dysfunction as assessed by the VEMP test has been shown to co-exist with normal semicircular canal responses in patients with connexin-26 mutations who do not complain of dizziness or unsteadiness [36] and in some patients with Meniere's disease who have normal caloric responses [37,38]. Given that normal semicircular canal function as assessed by clinical vestibular testing can not predict saccular function, the VEMP evaluation was extended to individuals with heterozygous *WFS1 *mutations and LFSNHL in this study for the first time. No saccular dysfunction was detected in person 26 segregating the c.2054G>C *WFS1 *mutation as this individual generated a normal VEMP response in both ears. This finding is interesting given that wolframin is expressed in vestibular hair cells [12] of the saccule (Guy van Camp, personal communication, 2007).

LFSNHL has also been associated with endolymphatic hydrops. An 8-year-old boy with LFSNHL and a *DIAPH1 *mutation was evaluated with EcochG and found to have an elevated SP/AP ratio, suggestive of endolymphatic hydrops [22]. To determine if abnormal EcochG findings were a trait of other dominant LFSNHL mutations, we provided two individuals segregating the c.2054G>C *WFS1 *mutation with an EcochG evaluation. The SP/AP ratio was normal for both persons 20 and 26, which suggests the absence of endolymphatic hydrops in these two individuals. It would be interesting to follow-up with the young man segregating the *DIAPH1 *mutation and other individuals in his family affected by LFSNHL to determine if an elevated EcochG SP/AP ratio is characteristic of the *DIAPH1 *mutation.

## Conclusion

We identified a small Caucasian American family presenting with hereditary autosomal dominant LFSNHL. Genetic analysis of the family revealed a heterozygous c.2054G>C *WFS1 *mutation that cosegregates with the hearing loss found in the family. This mutation, absent in 230 control chromosomes, predicts a non-conservative p.R685P substitution. For the first time, we describe VEMP and EcochG findings for individuals segregating a heterozygous *WFS1 *mutation, with both diagnostic tests yielding normal findings in this family.

## Abbreviations

LFSNHL: low frequency sensorineural hearing loss; VEMP: vestibular evoked myogenic potential; EcochG: electrocochleography; *WFS1*: Wolfram syndrome type 1 gene; *DFNA*: autosomal dominant deafness locus; *DIAPH1*: *diaphanous*; *MYO7A*: myosin VIIA (*MYO7A*); PCR: polymerase chain reaction; SP: summating potential; AP: action potential; LOD: log of the odds; dB: decibels; nHL: normalized hearing level.

## Competing interests

The authors declare that they have no competing interests.

## Authors' contributions

NFB elaborated the design of the study and conducted the DNA sequence analysis. JCK performed the microsatellite marker analysis. AMV investigated EcochG protocols and modified the consent forms appropriately. VAS collected blood samples and audiograms.

## Pre-publication history

The pre-publication history for this paper can be accessed here:


